# Plants with Potential Importance in Supporting the Treatment of Depression: Current Trends, and Research

**DOI:** 10.3390/ph17111489

**Published:** 2024-11-06

**Authors:** Renata Nurzyńska-Wierdak

**Affiliations:** Department of Vegetable and Herb Crops, Faculty of Horticulture and Landscape Architecture, University of Life Sciences in Lublin, Doświadczalna 50a, 20-280 Lublin, Poland; renata.nurzynska@up.lublin.pl

**Keywords:** mental disorders, herbal medicines, antidepressants, mechanisms of action

## Abstract

Depression is one of the most common diseases in the world, and it is also the most common mental disorder. Depressive disorders are a real threat not only to individuals, but also to the general population. This disease is a leading cause of disability and inability to work. Due to the numerous side effects of conventional drugs, attention is increasingly being paid to other solutions, including herbal medicines. Many plant species are known for their traditional uses in the treatment of anxiety, insomnia, and depression. The clinically proven effects of adaptogenic raw materials on depression symptoms are probably related to the positive impact of some secondary metabolites (terpenoids, alkaloids, glucosinolates, phenols). Currently, it is emphasized that in many cases the antioxidant and anti-inflammatory properties of plant substances play a protective role at the neurocellular level. Among the medicinal plants analyzed in clinical trials for the treatment of depression, the following seem to be particularly interesting: saffron (Crocus L.), turmeric (Curcuma L.), ginkgo (Ginkgo L.), St. John’s wort (Hypericum L.), and passionflower (Passiflora L.), which have broad and strong biological activity, well-documented history of action and use, and effectiveness in preventing and/or treating anxiety and depression. These plants are still in the sphere of biochemical and phytopharmaceutical research, the results of which are very promising.

## 1. Introduction

Depression is a common mental illness with severe consequences for human performance. Various factors are involved in the development of depression, with biological and social factors being among the most prevalent [1]. Biological theory emphasizes the significant role of genetic predisposition, changes at the level of neurotransmitters and hormonal systems, and the localization of changes in specific areas of the brain in the occurrence of depressive disorders. More recently, it has been reported that intrinsic stressors such as changes in serum levels of cholesterol, triglycerides, glucose, and clotting factors are associated with depression. Depression is thought to arise from a complex interaction between biological, social, psychological, and epigenetic factors [2]. The goal of treating depression is primarily to achieve symptomatic remission as wholly and quickly as possible and to prevent relapse. There are severe defects with most synthetic antidepressants, such as a narrow spectrum, side effects, high price, and easy relapse [3,4]. Serious side effects of synthetic antidepressants and antianxiety drugs include headaches, sexual dysfunction, addiction, seizures, and suicide attempts. The side effects mentioned above can be reduced by using appropriate herbal medicines with explicit activity in the nervous system [4,5,6]. Many patients prefer herbal medicines because of the side effects and destructive effects of chemical drugs. Research is focused on finding multidirectional antidepressants with low toxicity [7,8].

The effects of some plant substances in cases of mild anxiety have been demonstrated mainly in studies with animal models, and the structure–activity relationships, metabolism, absorption, and neuropharmacological mechanisms are mostly unclear. Modulating the GABA (gamma-aminobutyric acid) system is likely a critical mechanism for this effect [6]. GABA, a major inhibitory neurotransmitter in the central nervous system (CNS), plays an indispensable role in the experience of anxiety. Anxiety and related neurological disorders often result from low levels of GABA in the CNS [9]. The antidepressant effect of most plants, moreover, involves increasing serotonin, norepinephrine, or dopamine levels in the brain [10]. A particular group of plant raw materials that show positive effects on human physiology in stressful situations are adaptogens. The term adaptogen was first proposed in 1940 by Nikolay Lazarev, who described *Schisandra chinensis* (Turch.) Baill. and other herbs non-specifically enhance the human body [11]. The clinically proven effects of adaptogenic raw materials on symptoms of depression are probably related to the positive effects of specific secondary metabolites (terpenoids, alkaloids, glycosinolates, phenols) on cellular allostasis [11,12]. It is now emphasized that in numerous instances, adaptogen-derived substances’ antioxidant and anti-inflammatory properties play a protective role at the neurocellular level [13].

## 2. Purpose of the Work

Medicinal plants and their active ingredients are constantly being researched, tested, and then trialed for the treatment of various diseases, including depression. Although most of these studies are preclinical, they remain preliminary, as the understanding of the mechanism of antidepressant action of many drugs and plants is still very inconclusive. The extensive literature on ethnopharmacological research indicates the great potential of plants used in the traditional treatment of depression and anxiety, which provides the opportunity for intervening studies to confirm their efficacy [13]. Various plant species have been described in the world literature as effective for depression [3,14,15,16,17]. Motti and de Falco [18] report that in Italy, plants belonging to 76 genera and 32 families are used as traditional sedatives and sleeping aids, with the most significant number in the Asteraceae (24.2%) and Lamiaceae (21.1%) families. Leaves (29%) and flowers (27%) are the most commonly used raw materials in the form of infusions (70%) and decoctions (25%). The authors mention the use of such species as *Anthemis arvensis* L., *Clinopodium nepeta* L., *Crataegus monogyna Jacq*, *Humulus lupulus* L., *Laurus nobilis* L., *Lavandula angustifolia Mill*., *Matricaria chamomilla* L., *Ocimum basilicum* L., *Papaver rhoeas* L., *P*. *somniferum* L., *Rosmarinus officinalis* L., *Tilia platyphyllus Scop*., and *Valeriana officinalis* L., stressing the need for research to clarify all the mechanisms of action of bioactive compounds and confirm their therapeutic potential.

In many cases, plants known for traditional uses have not yet been sufficiently studied, nor have their activity and efficacy been confirmed. Plants included in research programs are still a source of scientific inspiration. Their rich chemical composition and the possibility of synergistic actions create new opportunities for their practical use. Among the medicinal plants analyzed in clinical trials for the treatment of depression, saffron (Crocus L.), turmeric (Curcuma L.), ginkgo (Ginkgo L.), St. John’s wort (Hypericum L.), and passionflower (Passiflora L.) are seemingly particularly intriguing. The plant species mentioned above exhibit broad and potent biological activity and have a well-documented history of action, use, and efficacy in the prevention and/or treatment of anxiety and depression. These plants are still in the realm of biochemical and phytopharmaceutical research. The remainder of this article presents critical information about the origins and traditional uses of the listed plant species with recognized CNS activities, scientific confirmations of their efficacy, and potential applications in the treatment of depression.

## 3. Phytopharmaceutical Characteristics of Selected Plant Species

### 3.1. Crocus sativus L. (Iridaceae Juss.)

Saffron provides a valuable spice and medicinal raw material: the dried stigma and the upper part or top of the pistil (*Croci stigma*) are the important parts of the flower. The primary producers of saffron are India, Iran, Morocco, Spain, and Greece. The history of the spice dates back 3000–4000 years and is associated with several continents and civilizations. Saffron is found in Greco-Roman, Egyptian, and Persian cultures and civilizations. From Persia, it spread to India and China, and around the 9th century, it appeared in Europe and worldwide [19]. Saffron is an essential medicinal resource and is considered a panacea in its countries of origin. In addition to its antidepressant effect, it has anticancer, expectorant, anticonvulsant, antimicrobial, and antioxidant effects (Table 1). The antidepressant effect of saffron is to increase serotonin and dopamine concentration in the central nervous system, resulting in improved mood. The antidepressant and neuroprotective effects of saffron are likely related to its anti-inflammatory and antioxidant activities [19,20].

The raw material of saffron contains more than 150 aromatic volatile compounds and many non-volatile active ingredients, such as carotenoids. The main biologically active components are crocin, crocetin, picrocrocin (a precursor of safranal), zeaxanthin, lycopene, α- and β-carotene—orange carotenoid pigments, flavonoids, and safranal, a volatile compound formed during drying of the raw material which provides it with its characteristic aroma [19,21]. Crocin and crocetin are essential active constituents of saffron (Table 1). Crocin is a rare carotenoid found in nature. It is readily soluble in water and has the most significant effect on the coloring power of saffron. Compared to other carotenoids, it is more widely used as a natural food and drug colorant, mainly due to its high solubility [21]. Crocin is poorly absorbed, and its excretion rate is relatively high. After oral administration, it is metabolized to crocetin. Crocetin, an aglycone of crocin, is rapidly absorbed and distributed to extravascular tissues after oral administration [22]. The substance can cross the blood–brain barrier and reach the central nervous system. For these reasons, it may be effective in neurodegenerative disorders. It should be noted that crocetin is also excreted from the body in significant amounts. Various nanotechnology solutions are currently being evaluated to improve the stability and bioavailability of crocin and crocetin [19].

The results of preclinical and clinical studies strongly suggest that saffron and its phytochemical compounds, particularly crocin, crocetin, and safranal, reduce the severity of depression [35,36,37]. The mechanism of saffron’s antidepressant effects has only been studied in recent decades [38,39]. These effects are likely related to saffron’s antioxidant, anti-inflammatory, and neuroprotective effects or its ability to modulate the levels of neurotransmitters in the brain, particularly serotonin, dopamine, glutamate, and GABA [22]. Wang et al. [40] report that the antidepressant properties of aqueous saffron extracts are related to the presence of crocin. Crocin exhibits significant antidepressant activity. After pretreatment with rapamycin, the antidepressant effect of crocin was significantly inhibited. It suggests that the mechanism of crocin’s antidepressant effect may be related to the mammalian target of the rapamycin (mTOR) signaling pathway [41]. Crocin had a significant antidepressant effect in a chronic corticosteroid-induced depression model in rats, suggesting that inhibition of inflammation and oxidative stress is associated with antidepressant effects [24]. Crocin supplementation at a daily dose of 30 mg for 8 weeks may alleviate depressive symptoms accompanying metabolic syndromes, and this effect seems to be independent of changes in plasma PAB concentration [42]. In contrast, Karimi et al. [43] found that the main components involved in the antidepressant effects of *C. sativus* are flavonoids and anthocyanins. The effect of saffron in the treatment of mild depression is similar to the synthetic antidepressants fluoxetine and imipramine [21]. Combined treatment with saffron and curcumin is associated with a more remarkable improvement in depressive symptoms in patients suffering from major depressive disorder compared to placebo [19]. It should be mentioned that saffron taken in large doses may increase the risk of miscarriage, so pregnant women should not use it without consulting a doctor. It is also not recommended to give saffron to children. Overdose can cause vomiting, diarrhea, and gastrointestinal and genitourinary bleeding [19,21,44].

### 3.2. Curcuma longa L. (Zingiberaceae Lindl.)

Turmeric is a well-known spice and medicinal plant. The rhizome of this plant (*Curcumae rhizoma*) is used as a spice and a safe remedy against various ailments in many countries (mainly in China and India). Turmeric has valuable medicinal properties: it has hepato-protective, choleretic and cholagogic, anti-inflammatory, antimicrobial, antioxidant, anticancer, and neuroprotective effects. It is essential in preventing and treating many disorders, particularly related to its antioxidant, anti-inflammatory, and antitumor effects. Its potential importance in treating and preventing diabetes is highlighted [45,46,47].

The turmeric rhizome contains 3% curcuminoids (dicynamoylmethane derivatives) and 3% essential oil containing mainly sesquiterpenes. Curcuminoid compounds include curcumin (C_21_H_20_O_6_), demethoxy-curcumin, and bis-demethoxy-curcumin, which account for about 77%, 17%, and 3% of the dry weight, respectively [45,48]. There are many publications describing the beneficial role of curcumin in the prevention and treatment of various chronic diseases (such as type 2 diabetes, Alzheimer’s disease, multiple sclerosis, atherosclerosis, and cancer) [45,49], which is due to its broad biological activity (Table 2).

Curcumin research has been ongoing since 1815 and has become increasingly important [47]. Recently, interest in curcumin as a treatment for psychiatric conditions has increased. There are a growing number of preclinical and clinical studies evaluating its antidepressant and antianxiety effects. The substance exhibits a wide range of pharmacological properties and has been recognized as a potent antidepressant with diverse mechanisms of action [54]. Curcumin’s multiple mechanisms of action provide a unique advantage in the treatment of depression, especially in terms of side effects. Compared to curcumin monotherapy, integration with other antidepressants seems to provide better antidepressant effects. It is indicated that future research should focus on combination antidepressant therapy [48].

The antidepressant effect of curcumin may be due to increased serotonin, norepinephrine, and dopamine levels in the brain. This substance may be a helpful antidepressant, especially in cases that respond to drugs with mixed effects on serotonin and catecholamine levels in the brain [55]. Curcumin is active in modulating neurotransmitter concentrations and inflammatory pathways, with beneficial effects in pathological processes leading to neuronal damage, hypothalamic–pituitary–adrenal disorders, insulin resistance, and oxidative stress, as well as operating within the endocannabinoid system [48,54]. The actions mentioned above may be involved in the pathophysiology of major depressive disorder (MDD). Several clinical trials have been published that suggest the benefits of curcumin in MDD [56,57]. Yaikwawong et al. [58] showed that curcumin supplements exhibit potential antidepressant effects on type 2 diabetes patients with obesity by elevating serotonin levels, reducing inflammation, and mitigating oxidative stress. With evidence gradually mounting, curcumin appears to be a promising alternative for treating MDD [48,59]. The endocannabinoid system (ECS) is a neuromodulator system that significantly affects the central nervous system (CNS) and the inflammatory response to endogenous and exogenous compounds. The ECS also affects anxiety, eating behavior, emotional responses, hypothalamic–pituitary–adrenal (HPA) axis activity, and neurogenesis—the formation and differentiation of new cells in neural tissue. Changes in the ECS have been identified in depression and represent a therapeutic target for intervention. In animal studies, the administration of curcumin had a beneficial effect on the ECS [60]. Depending on the type of depression (acute or chronic), the duration of monitoring, and the doses used, curcumin has a positive effect on reducing depressive symptoms, with a possible mechanism of action involving different pathways. Its effects are mainly associated with antioxidant, anti-inflammatory, and neuroprotective properties [61]. Evaluation of curcumin’s efficacy in various inflammatory psychiatric disorders, such as schizophrenia, depression, and autism, has proven a positive effect in reducing psychiatric deficits [59,62].

One of the main problems with curcumin’s applicability is its poor bioavailability and instability. Curcumin is unstable at physiological pH and is rapidly degraded by autooxidation reactions. Alkaline hydrolysis products (ferulic acid, vanillin, ferulic aldehyde, and feruloylmethane), as well as oxidation products (such as bicyclopentadione), show biological activity but are much less active than curcumin [47]. It has been proven that curcumin’s anti-inflammatory effects are mediated by its oxidative metabolites [50]. Orally administered curcumin is poorly absorbed in the small intestine, rapidly metabolizes, and is eliminated from the body. To overcome these difficulties, several curcumin preparations have been developed in the form of nanoparticles or nanoemulsions [47]. Carvalho et al. [63] demonstrated the effectiveness of reducing curcumin’s particle size in increasing solubility. Preparing the nanosuspension did not degrade the curcumin particles, and the nanosuspension proved to be more soluble than the usual mixture. The reduction in particle size did not improve the antioxidant activity of curcumin. Pure curcumin and the tested formulations showed satisfactory antioxidant activity.

Human studies have shown no toxic effects of curcumin administered orally 6 g/day for 4–7 weeks [47]. The substance is well-tolerated, and the frequency of reported side effects can be compared with placebo. The most common side effects include mild gastrointestinal discomfort, such as nausea, mild abdominal pain, and diarrhea. Clinical trials to date have lasted from 4 to 16 weeks, with treatment doses varying widely. The optimal treatment doses have yet to be determined [59]. The results confirm that curcumin is a promising drug for treating depression, but further research in this area is needed. Curcumin may become a promising alternative to many current high-risk treatments as a natural, accessible, and safe compound [60].

### 3.3. Ginkgo biloba L. (Ginkgoaceae Engl.)

Ginkgo is a long-lived endemic and relict species that occurs naturally only in China. It has long been known in traditional Chinese medicine [64]. Abundant in polyphenols, ginkgo leaves (*Ginkgo bilobae folium*) are a raw material more commonly used to prevent neurological, cardiovascular, or hyperglycemic diseases [65]. The main active constituents of ginkgo leaves are flavonoids (ginkgo-flavonoid glycosides), terpenoids (ginkgolides and bilobalides), biflavones, and organic acids. Ginkgolides, unique to ginkgo, are not synthesized by other living species. These compounds are classified as types A, B, C, J, or M. Flavonoids, such as quercetin, kaempferol, and isorhamnetin, occur as glycoside derivatives [66,67]. Dried ginkgo leaves are currently used to obtain standardized aqueous or ethanol extracts. Ginkgo extracts (GEs) are used to treat cerebrovascular disorders, dementia, and memory impairment, as well as to combat symptoms associated with mild to moderate dementia and in combination therapy with antidepressants [61,65]. Pharmacological studies of GE mainly focus on determining the effects of the whole extract, separated fractions, or single active compounds on the nervous and cardiovascular systems. The broad activity of the extracts is due to the synergistic action of all its components: flavonoids, terpenoids, organic acids, and others. Ginkgo ethanol extracts contain about 22–27% flavonol glycosides, including polyphenols such as tannins and terpene lactones (5–7%). High phenolic content determines intense iron ion activity, antioxidant power, copper chelating ability, and superoxide radical scavenging activity [68]. Kobus-Cisowska et al. [69] showed a positive correlation between the content of total flavonols (r = 0.632), total phytosterols (r = 0.711), and activity against DPPH radicals, as well as the content of α-tocopherol (r = 0.891) and ABTS. Flavonols such as rutin, quercetin, morin, and kaempferol significantly affected antioxidant activity against DPPH radicals, but the highest correlation coefficient was found for myricetin (r = 0.812).

GEs exhibit a wide range of biological activities (Table 3), with antioxidant activity being their primary activity. The chemical composition of GEs and their antioxidant activity depends on the date of leaf harvesting and the type of solvent used [69]. Klomsakul et al. [70] demonstrated by the DPPH method the antioxidant properties of GE at a concentration of 500 µg mL^−1^ at 95.29%, comparable to BHT, ascorbic acid, and gallic acid used as positive controls. The antioxidant activity of *G. biloba* has been linked to several therapeutic effects, and GEs are currently indicated for the treatment of vagus inflammation, headache, memory disorders, stoppage chroma, dementia, Alzheimer’s disease (AD), glaucoma, cardiovascular disorders, cerebral ischemia, increased libido and sexual activity, and psychiatric diseases such as depression [71]. Research by Bogacz et al. [72] suggests that GE may modulate the expression of cytochrome P450 enzymes, and transcription factors may be involved in the metabolism of xenobiotics (drugs, procarcinogens, vitamins, food ingredients). Suárez-González et al. [73] showed that kaempferol, quercetin, and luteolin have multidirectional potential against Alzheimer’s disease and exhibit antioxidant activity against reactive oxygen species (ROS). GEs act multidirectionally by inhibiting enzymes responsible for neurotransmitter degradation and amyloid plaque formation, reducing ROS in the CNS, decreasing its degradation, and promoting amyloid plaque formation.

GE has a long history of clinical applications in treating brain and psychiatric disorders, but the critical mechanism remains incompletely understood. Antioxidation, anti-inflammatory effects, anti-apoptosis, defense against mitochondrial dysfunction, amyloidogenesis, and Aβ aggregation, modulation of tau protein phosphorylation, ion homeostasis, and even induction of growth factors are possible mechanisms of action of *G. biloba* [80]. Ginkgo active constituents control psychiatric disorders by regulating neurobiological mechanisms [81]. Hasanvand and Farhadi [82] suggest that physical activity and *G. biloba* supplementation can improve brain function in older people with depression. Dai et al. [78] confirmed the effectiveness of using GE in the supportive treatment of elderly patients with depression. The extract restores neurological function, and when combined with antidepressants, it plays a synergistic role (the effect is faster than with single antidepressants). The neuroplasticity hypothesis of depression suggests that a lack of brain-derived neurotrophic factor (BDNF) may cause structural changes in the brain. Pretreatment with 30 and 60 mg/kg/day of GE significantly attenuated depressive effects in rats compared to control groups. Various effects were analyzed; for example, 30 and 60 mg/kg of GE reduced the immobility time of rats in the forced swim test compared to stressed rats. Immobility time significantly increased in stressed and experimental groups treated with 15 mg/kg of GE, as compared to control rats, the overall effect size f = 2.2. In addition, it inhibited chronic-stress-induced changes in hippocampal BDNF DNA methylation and protein expression. These studies suggest that GE may play an antidepressant role by modulating BDNF expression in the hippocampus [83]. Kalkunte et al. [74] found that lipophilic ginkgo leaf extracts (LEGs) at 50 and 100 mg/kg, p.o. exhibited dose-dependent significant antidepressant activity comparable to imipramine and EGb 761 (a standardized extract of dried ginkgo leaves containing 24% flavonol glycosides, 6% terpene lactones such as ginkgolides A, B, C, J, and bilobalide). LEGs were rich in the 6-salicylate alkyl group (6-AS), along with fatty alcohols, fatty acids, and cardanols, without dihydroxyalkylphenols that cause allergic reactions. In commercial Ginkgo products, these dihydroxyphenols, as well as the beneficial 6-AS, are removed during the enrichment of flavonol glycosides and terpene lactones. However, it turns out that intact carboxylic acid groups containing 6-AS are bioactive components of lipophilic ginkgo leaf extract with antidepressant and antistress effects. Zhou et al. [84] demonstrated that GE exerted antidepressant effects in mice by regulating gut microbial metabolism. The potential involvement of *Parasutterella exkrementihominis* and a metabolite of ginkgolic acid, ursodeoxycholic acid, was revealed. The authors suggest that GE acts on bile acid metabolism modulated by the gut microbiome to alleviate stress-induced depression. This study explains a novel mechanism of GE in improving depression-like behavior in mice from the gut microbiota perspective and regulation of bile acid metabolism.

It is noteworthy that the efficacy of ginkgo preparations is emphasized, as is their safety [66,78]. GE has been shown to have low toxicity with long-term or acute administration, with no mutagenic or teratogenic effects [85], as well as an immune-enhancing effect at non-toxic concentrations on human peripheral blood leukocytes [68]. Currently, several phytotherapeutic products with *G. biloba* are available on the market with indications for disorders and symptoms associated with blood flow disorders in the brain [71]. Szwajger et al. [86] have shown that commercial enzyme preparations (names and detailed compositions of preparations are available from authors) exhibited very differentiated anticholinesterase and antioxidant activities. Some preparations were very poor sources of cholinesterase inhibitors and phenolic antioxidants. The authors suggest that differences in results can be caused by the various content of phenolic compounds, and the identification of the main constituents responsible for the anticholinesterase activity of preparations produced from *G. biloba* leaves is a priority.

### 3.4. Hypericum perforatum L. (Hypericaceae Juss.)

**Figure 1:**
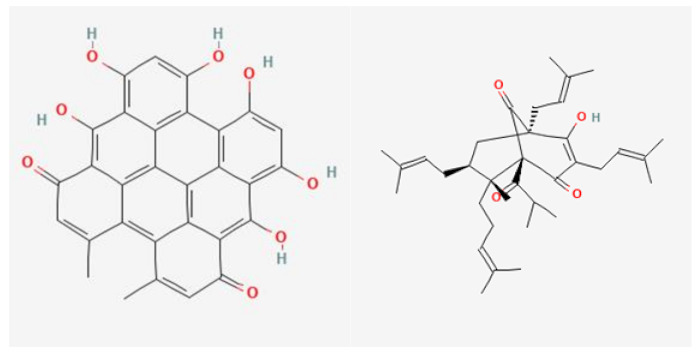
Structure of hypericin and hyperforin, active substances of SJW.

St. John’s wort (SJW) is traditionally used as an anti-inflammatory, diuretic, sedative, and mood-enhancing agent to treat wounds, ulcers, and metabolic disorders. Infusions of SJW herb (*Hyperici herba*), which contain mainly flavonoids and phenolic acids, are traditionally used in digestive system diseases and liver as choleretic and cholagogic preparations. Alcoholic extracts of SJW herb, on the other hand, show sedative and antidepressant effects, which have been clinically confirmed. SJW, especially its two main active constituents, hyperforin and hypericin (Figure 1), has been shown to have therapeutic effects on various mental and mood disorders, such as post-traumatic stress disorder (PTSD), attention deficit hyperactivity disorder (ADHD), obsessive-compulsive disorder (OCD), and anxiety disorders. SJW herb has also been shown to have antidepressant effects [87]. It is indicated that for patients with mild to moderate depression, SJW herb shows comparable efficacy and safety to SSRI (selective serotonin reuptake inhibitor) drugs [87,88].

The mechanism of SJW antidepressant action is not fully understood. The effect has been linked to hypericin and hyperforin, compounds that regulate levels of essential neurotransmitters such as dopamine, serotonin, norepinephrine, and GABA. Recent work indicates that hyperforin has a more potent antidepressant effect than hypericin, as it is a more potent inhibitor of serotonin, norepinephrine, dopamine, GABA, and glutamine reuptake. Hyperforin, isolated from SJW herb, contributes to the extract’s effects on excitability and neurotransmission. The compound increases extracellular serotonin levels [3]. The biological efficacy of SJW herb in treating depression is most likely due to the synergistic effects of the various components of the raw material, acting both within the central nervous system and peripherally. SJW has been compared favorably with numerous antidepressants, with studies showing equivalent results and a significantly lower incidence of side effects. In addition, its efficacy in treating seasonal affective disorder has been proven [89]. The use of SJW (due to its low incidence of side effects) can be of great benefit to patients with psychiatric or mood disorders. Szegedi et al. [90] evaluated the effect of *Hypericum* extract (HE) in treating moderate or severe unipolar major depression. The authors used 900 mg/day HE, a hydroalcoholic extract from *Hyperici herba* (drug to extract ratio 3–7:1) with standardized contents of 3–6% hyperforin and 0.12–0.28% hypericin. The coated tablets contained 300 mg or 600 mg of the extract. The patients (251 adult outpatients with acute major depression) were given HE three times a day or 20 mg paroxetine, one of the leading synthetic antidepressants, once a day for six weeks. In initial non-responders, doses were increased to 1800 mg/day hypericum or 40 mg/day paroxetine after two weeks. Ultimately, the authors concluded that in the treatment of moderate to severe major depression, HE is at least as effective as paroxetine, and is better tolerated. Indications for the use of SJW include mild depressive states [91]. It should be noted that SJW has a significant potential for drug interactions. Adverse reactions are mainly due to drug interactions with other selective serotonin reuptake inhibitors, opiates, and some antidepressants, cold and flu medications, which may lead to serotonin syndrome [92].

### 3.5. Passiflora incarnata L. (Passifloraceae Juss. ex Kunth in Humb)

*P. incarnata* L. is an evergreen vine that grows up to 6 m. The species is native to North and South America, Australia, and Southeast Asia and is now cultivated for its raw material for pharmaceutical purposes. Flowers, leaves, and seeds are widely used parts of this plant for medicinal purposes [93,94]. In Native American medicine, passionflower was used in particular by the Cherokees of the southern Allegheny Mountains, the Houmas of Louisiana, and the Aztecs of Mexico. Ancient Colombians traditionally used the plant as a sedative to treat insomnia and nervousness [93]. *P. incarnata* is one of the best-documented species of the Passiflora genus with medicinal properties. The plant’s above-ground parts, flowers, and fruits are used for medicinal purposes and are attributed to anthelmintic, antispasmodic, and antianxiety effects. The plant is also used as a remedy for burns, diarrhea, painful menstruation, hemorrhoids, neurotic disorders, insomnia, the treatment of morphine addiction, convulsions, or neuralgia [95]. Research recently confirms the broad biological activity of passionflower. Ożarowski et al. [96] showed that crude leaf extracts of *P. alata*, *P. caerulea*, and *P. incarnata* exhibited not only in vitro antioxidant potential but also antibacterial, antifungal, amoebostatic, and amoebicidal activities.

Passiflora contains several active substances, including alkaloids, phenolic acids, flavonoids, tannins, sterols, carotenoids, and cyanogenic compounds (Table 4). The primary chemical constituents of the *P. incarnata* flower are flavonoids (0.25%) and indole alkaloids (0.1%) based on the betacarboline ring system [94,97,98]. Passionflower leaf extracts contain significant amounts of polyphenols—phenolic acids, flavonoids, and tannins (2.48 mg gallic acid equivalent/g extract, 2.10 mg/g, and 1.9 mg/g, respectively)—and have high radical scavenging activity. The highest accumulation of flavonoids occurs in the leaves, with the highest concentration of isovitexin between the pre-flowering and flowering phases [99]. Guseinov et al. [100] determined 20 flavonoid-like compounds in the dry extract of *P. incarnata* herb. The total content of flavonoids per vitexin was 3.762 ± 0.049%, and that of vitexin was 0.867 ± 0.011%. Studies on the seeds of *P. incarnata* proved their usefulness due to the presence of β-sitosterol [101].

*P. incarnata* is used to treat anxiety and insomnia. A fraction derived from the methanolic extract shows significant antianxiety activity in mice. Passionflower extract may help treat absence seizures, which may be related to its effects on the GABAergic and opioid systems [105]. A methanolic extract of *P. incarnata* leaves showed significant sedative, anticonvulsant, and depressant effects on the CNS at 200 mg/kg doses in mice. The extract also showed analgesic and anti-inflammatory effects [106].

Herbal medicines derived from *P. incarnata* are used to treat some CNS disorders. The plant is used to treat insomnia and anxiety disorders in Brazil, Europe, and the US [3]. Jafarpoorh et al. [107] showed that a hydro-alcoholic extract of passionflower has significant antidepressant effects in animal models of depression. The authors recommend the simultaneous use of serotonin and GABA antagonists along with passionflower extract, as well as isolating the extract’s components and evaluating their effects on depression to determine the exact mechanism of antidepressant action. Santosh et al. [108] evaluated the effects of a methanolic extract of *P. foetida* leaves on depression in mice. The extract was shown to have specific antidepressant effects in vivo. The authors suggest that *P. foetida* leaf extract has potential antidepressant activity that may have therapeutic value in treating patients with depressive disorders. German physicians evaluated passionflower extract as effective in improving immunity and quality of life in patients suffering from nervous anxiety and well-tolerated (three cases of mild adverse events—fatigue—were reported) [109]. Blecharz-Klin et al. [110] conducted behavioral tests on rats provided *P. incarnata* extract (30, 100, 300 mg/kg) to elucidate the neurobiochemical basis of behavioral changes. The study indicated that exposure to the extract resulted in a dose-dependent modulation of the rats’ behavior and altered neurotransmission in significant components of the CNS motor system. The changes primarily affected the cerebellar and spinal dopaminergic and noradrenergic systems. In animals receiving *P. incarnata* extract, the concentration of 5-hydroxyindoleacetic acid in the cerebellum, the structure of the CNS involved in maintaining balance and the coordination of movements, was lower compared to the control group. Significant differences were found also in the concentration of homovanillic acid in the medulla oblongata (F(3.36) = 2.68; *p* < 0.05) and the spinal cord. Significant changes in the concentration of GABA were found in the cerebellum (F(3.36) = 3.56; *p* < 0.05) and the spinal cord (F(3.36) = 6.71; *p* < 0.005). In the cerebellum, the concentration of histidine was higher in the group receiving *P. incarnata* extract at the highest dose compared to the other groups (F(3.36) = 3.44; *p* < 0.05). The authors conclude that due to its inhibitory effect on the GABAergic pathway, passionflower extract has potential as a sedative and may be an alternative to benzodiazepines in controlling anxiety and hyperactivity. However, further research is needed to determine the molecular mechanism of action of *P. incarnata* extract and its effects on neurotransmitter pathways in central nervous system structures and motor functions. Jawna-Zboińska et al. [111] conducted a study to evaluate the behavioral and neurochemical effects of long-term oral administration of *P. incarnata*. They tested passion flower extract (30, 100, or 300 mg/kg body weight/day) administered to 4-week-old male Wistar rats with drinking water. Tests were conducted after seven weeks of treatment. Reduced anxiety and dose-dependent improvements in memory were observed in rats given passiflora compared to the control group. The experimental groups had significantly different concentrations of serotonin in the prefrontal cortex (F(3.36) = 5.04, *p* < 0.005), hippocampus (F(3.36) = 2.68, *p* < 0.05), hypothalamus (F(3.36) = 2.56, *p* < 0.05), and striatum (F(3.36) = 6.42, *p* < 0.005). The authors demonstrated that passionflower affects serotonergic and glutamatergic neurotransmission in brain structures responsible for memory processes and partially confirmed the mechanism of action of *P*. *incarnata* in relation to GABAA receptors, indicating the need for further research in this area [111].

*P. incarnata* is listed by the U.S. Food and Drug Administration as a “safe herbal sedative”, and none of the available *P. incarnata* monographs mentions toxicity or contraindications of this plant. Chikhale et al. [112] report that the administration of passionflower was not associated with any side effects, such as memory loss or a breakdown in psychometric abilities. Like oxazepam or midazolam, *P. incarnata* provides sedative effects. It appears to be a safe and effective drug for reducing stress reactivity, insomnia, anxiety, and depressive behavior. However, since the physiological effects and mode of depressant action on the CNS have not been well-documented, caution is advised when using *P. incarnata* together with other CNS depressants or stimulants [99].

## 4. Conclusions

Herbal medicines, known for their traditional health-promoting uses, are now being increasingly studied and trialed in combination therapies for depression. They are characterized by efficacy, synergism of action, and a high safety profile. Patients with depression often suffer from cognitive impairment, probably as a result of oxidative stress. Many herbal medicines are well-known for their antioxidant and anti-inflammatory activities and may alleviate symptoms of depression through the effects mentioned above. This work collects available information on both the traditional use of selected plants in depression and modern therapeutic solutions. The examples provided indicate the advisability of searching for new antidepressants among the traditional ones.

The rich chemical composition of plant medicines allows for versatile effects on the body. Active compounds may act on one or more target proteins that regulate neurotransmitter function, the HPA axis, the BDNF signaling pathway, the anti-inflammatory response, oxidative stress, gut microbiota, and ferroptosis. Herbal ingredients combined can presumably act on several mechanisms in a coordinated manner. Used properly, they appear to be effective in treating depression without causing adverse side effects. Herbal extracts have a long history of clinical applications in treating brain and mental disorders, but the critical mechanism remains incompletely understood in most cases. This creates the need for further intensive research into the activity of individual plant substances and evaluations of possible synergistic or antagonistic effects, mechanisms of action, and pharmacokinetics.

## Figures and Tables

**Table 1:** Main bioactive constituents of saffron.

Compound/Structure	Biological Activity	Information Sources
Crocin 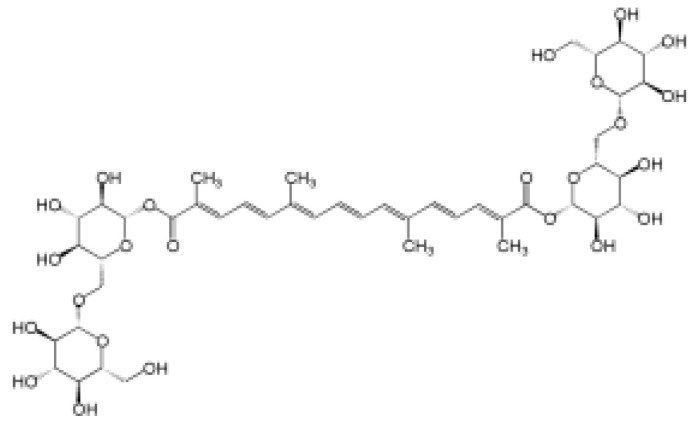	Antioxidant, anticancer, antidepressant, neuroprotective related to memory and dementia	[21,22,23,24,25,26]
Crocetin 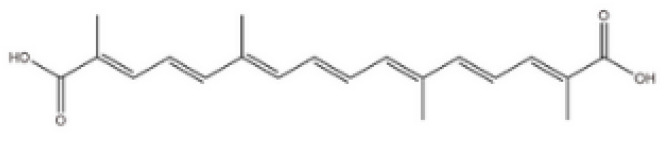	Antioxidant, cardioprotective, hepatoprotective, neuroprotective, antidepressant, antiviral, anticancer, atherosclerotic, antidiabetic, memory-enhancing	[23,27,28]
Safranal 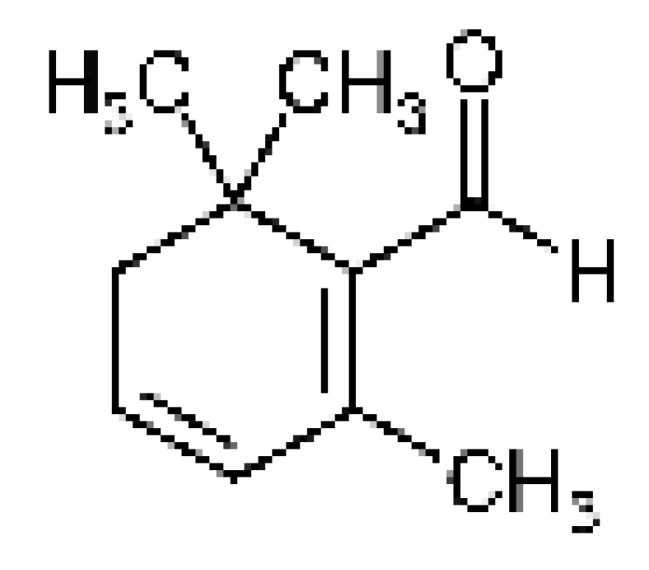	antioxidant, anti-inflammatory, antidepressant, anxiolytic, antiasthmatic, antihypertensive, anticonvulsant, anticancer, antitussive, antigenotoxic antibacterial, cardioprotective, neuroprotective, nephroprotective, gastrointestinal protective, immunoregulatory	[29,30,31,32,33,34]

**Table 2:** Biological activity of curcumin—the main biocomponent of turmeric.

Compound/Structure	Biological Activity	Information Sources
Curcumin 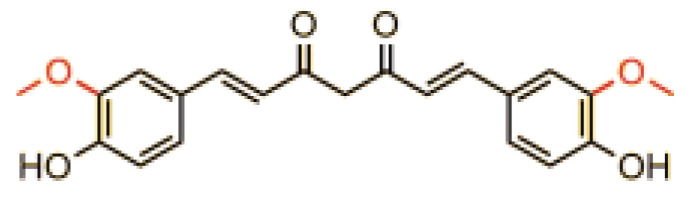	Anti-Alzheimer, antibacterial, anticancer, antidiabetic, antifungal, anti-inflammatory, antimalarial, antinociceptive, antioxidant, antiprotozoal, antiviral, wound healing	[46,47,49,50,51,52,53]

**Table 3:** Biological activity of GE.

Biological Activity	Information Sources
Anti-Alzheimer, anti-aging, anti-allergic antianxiety, antibacterial, antifatigue, anti-inflammatory, antioxidant, antistress, antiulcer, cardioprotective, cholinesterase inhibition, immunoregulatory, neuroprotective, tyrosinase, anti-DOPA auto-oxidation inhibition, and cytotoxic anticancer	[67,68,69,70,74,75,76,77,78,79]

**Table 4:** Main bioactive constituents of *P. incarnata*.

Compounds	Information Sources
Flavonoids	
Apigenin, chrysin, isoorientin, isoschaftoside, isovitexin, isovitexin-2″-O-β-glucoside, isoorientin-2”-O-β-glucoside, hyperoside, kaempferol, kaempferitrin, luteolin, orientin, quercetin, rutin, saponarin, schaftoside, scutelarein, vicenin, vitexin, tri-substituted benzoflavone compound (BZF)	[93,99,100,102,103]
Phenolic acids	
Chlorogenic acid, caffeic acid	[102]
Alkaloids	
Harman, harmol, harmine, harmalol, harmaline	[99]
Glycosides	
Byzantionoside B, foliasalacioside L, cyanogenic glycoside gynocardin, roseoside,	[103]
Others	
Amino acids, carbohydrates (raffinose, sucrose, D-glucose, D-fructose), essential oil, dehydrovomifoliol, γ-benzo-pyrone derivative maltol, uridine	[99,103,104]

## Data Availability

Data is contained within the article.
